# Homopolymer tract length dependent enrichments in functional regions of 27 eukaryotes and their novel dependence on the organism DNA (G+C)% composition

**DOI:** 10.1186/1471-2164-5-95

**Published:** 2004-12-14

**Authors:** Yue Zhou, Jeffrey W Bizzaro, Kenneth A Marx

**Affiliations:** 1 Division of Endocrinology, Gerontology, and Metabolism, Stanford University School of Medicine, Stanford, CA, USA; 2 Center for Intelligent Biomaterials, Department of Chemistry, University of Massachusetts, Lowell, MA, USA

## Abstract

**Background:**

DNA homopolymer tracts, poly(dA).poly(dT) and poly(dG).poly(dC), are the simplest of simple sequence repeats. Homopolymer tracts have been systematically examined in the coding, intron and flanking regions of a limited number of eukaryotes. As the number of DNA sequences publicly available increases, the representation (over and under) of homopolymer tracts of different lengths in these regions of different genomes can be compared.

**Results:**

We carried out a survey of the extent of homopolymer tract over-representation (enrichment) and over-proportional length distribution (above expected length) primarily in the single gene documents, but including some whole chromosomes of 27 eukaryotics across the (G+C)% composition range from 20 – 60%. A total of 5.2 × 10^7 ^bases from 15,560 cleaned (redundancy removed) sequence documents were analyzed. Calculated frequencies of non-overlapping long homopolymer tracts were found over-represented in non-coding sequences of eukaryotes. Long poly(dA).poly(dT) tracts demonstrated an exponential increase with tract length compared to predicted frequencies. A novel negative slope was observed for all eukaryotes between their (G+C)% composition and the threshold length N where poly(dA).poly(dT) tracts exhibited over-representation and a corresponding positive slope was observed for poly(dG).poly(dC) tracts. Tract size thresholds where over-representation of tracts in different eukaryotes began to occur was between 4 – 11 bp depending upon the organism (G+C)% composition. The higher the GC%, the lower the threshold N value was for poly(dA).poly(dT) tracts, meaning that the over-representation happens at relatively lower tract length in more GC-rich surrounding sequence. We also observed a novel relationship between the highest over-representations, as well as lengths of homopolymer tracts in excess of their random occurrence expected maximum lengths.

**Conclusions:**

We discuss how our novel tract over-representation observations can be accounted for by a few models. A likely model for poly(dA).poly(dT) tract over-representation involves the known insertion into genomes of DNA synthesized from retroviral mRNAs containing 3' polyA tails. A proposed model that can account for a number of our observed results, concerns the origin of the isochore nature of eukaryotic genomes via a non-equilibrium GC% dependent mutation rate mechanism. Our data also suggest that tract lengthening via slip strand replication is not governed by a simple thermodynamic loop energy model.

## Background

DNA homopolymer tracts are the simplest of simple sequence repeats (SSRs); the two types being poly(dA).poly(dT) and poly(dG).poly(dC). They are present in all genomes, but in some eukaryotes they are found at high frequencies indicating that the tracts are highly enriched relative to their random occurrence within a random sequence DNA genome of similar base composition. Homopolymer tracts were previously examined systematically in the coding, intron and flanking regions of the slime mold *D. discoideum *[1]. Only long (N>10 bp) homopolymer tracts were observed at high frequencies in this AT-rich genome. The non-coding regions were found to be highly over-represented in the large poly(dA).poly(dT) tracts compared to random sequences of equivalent base composition containing tracts at frequencies expected for random occurrence. At shorter sequence lengths (2 bp<N<6 bp), poly(dG).poly(dC) tracts were over-represented somewhat more than poly(dA).poly(dT) tracts of comparable length.

Although the elongation of SSR tracts may be due to more than one mechanism [2], most often the phenomenon has been attributed to slip-strand replication errors, which occur from the slippage and re-annealing of the nascent strand during DNA replication [3-5]. This is a type of mutation that can be affected by the proofreading function of DNA polymerases [6-8]. For example, it has been shown that the proofreading and repair function for DNA polymerase epsilon is efficient for short homopolymer tracts, but that only the mismatch repair system can prevent frameshift mutations in tracts of length 8 nucleotides or greater [8]. These slip-strand errors, which lead to the formation of longer homopolymer tracts, can have deleterious effects. In coding regions, the mutation can cause frame-shift errors, leading to transcription errors and aberrant protein translation. As would be expected from the triplet codon constraints, there appears to be selection against long homopolymer tracts in coding regions. Marx et al [1] demonstrated that long homopolymer tracts were not present at frequencies higher than expected in the coding regions of *D. discoideum *DNA.

Compared to nonhomopolymer random B-DNA sequences, poly(dA).poly(dT) tracts have a shorter turn, a smaller axial rise, a narrower and deeper minor groove [9], a wider and shallower major groove, and are straighter and more rigid over longer lengths. These characteristics are due to the high propeller twist of the base pairs [10], the maximal overlap/stacking between bases on the same strand, and non-Watson-Crick cross-strand H-bonds between base pairs [9-13]. The result is that long tracts tend to be energetically excluded from nucleosomes [14,15]. In some definitive studies, it was first shown that tracts of critical and longer lengths are excluded from the reconstituted nucleosome [16]. Other investigators, using native nucleosomes derived from native chicken chromatin, demonstrated that long poly(dA).poly(dT) tracts are excluded from the central core regions [17]. This conclusion received confirmation from a study of the GenBank sequences of *D. discoideum*, where it was demonstrated that long poly(dA).poly(dT) tracts (N>10 bp) are preferentially spaced at sequence lengths corresponding to the average nucleosome DNA spacing in *D. discoideum *nucleosomes [18]. In this study, adjacent long poly(dA).poly(dT) tracts, combined with their short nonhomopolymer spacer sequences, exhibited average total lengths that correspond to *D. discoideum *nucleosomal linker lengths, suggesting their *in vivo *localization in these chromatin regions and avoidance of the nucleosomal core regions.

Certain natural DNA sequences possess tertiary structures exhibiting a significant amount of curvature that is associated with short homopolymer lengths (4 – 6 bp) of poly(dA).poly(dT) [19,20]. Also, bending occurs at the junction of these and nonhomopolymer tracts [12]. When short bent poly(dA).poly(dT) tracts are distributed 10 bp apart, they produce additive long range in-plane bending in the axis of the DNA helix [20,21]. The exact molecular mechanism of this bending behavior is still the subject of considerable experimentation and speculation [22]. DNA bending patterns resulting from spaced poly(dA).poly(dT) tracts have been shown to occur in replication origins and in transcriptional regulatory regions, where a bent configuration is required for activity [23-25].

Poly(dG).poly(dC) tracts form an A-form double-helix. In contrast to poly(dA).poly(dT), the minor groove of these tracts is broad and shallow, while the major groove is deep. But, as with poly(dA).poly(dT) tracts, the tracts are rigid, which leads to the energetic exclusion of poly(dG).poly(dC) from nucleosomes [26,27]. These characteristics are due to the overlap of adjacent guanine bases and the invariant roll angle between them [28].

Beyond their structural properties, studies of homopolymer tracts have revealed some biological functions. The poly(dA).poly(dT) tracts can serve as protein binding sites [29], particularly as upstream promoter elements in the initiation of transcription [30-32] and in recombination [33]. And poly(dG).poly(dC) tracts have been found in certain eukaryotic promoter regions where they are postulated to form 4 stranded G-quartet structures [34].

As the number of DNA sequences publicly available increases, the representation (over and under) of homopolymer tracts in different genomes can be compared. Qualitative comparisons have been made between five eukaryotic and two prokaryotic genomes: *P. falciparum *(also very AT-rich), *H. sapiens*, *S. cerevisiae*, *C. elegans*, *A. thaliana*, *E. coli *and *M. tuberculosis *[35]. As with *D. discoideum *(1), it was shown in that study that homopolymer tracts occur in the non-coding regions at over-represented frequencies for poly(dA).poly(dT). Poly(dG).poly(dC) tracts were found to be over-represented in some but not all organisms at short lengths. However, over-representation was observed only in the eukaryotic genomes, not the prokaryotes.

In the present study, we have carried out a broad survey of non-overlapping homopolymer tract frequencies in the genomic sequences of 27 eukaryotic organisms across the base composition range from 20–60% (G+C). Within the coding, intron and flanking DNA functional compartments of largely single copy genes from these organisms, we compared the observed poly(dA).poly(dT) and poly(dG).poly(dC) tract frequencies in two size ranges to the tract occurrence frequencies expected for random tract occurrence in DNA compartments of the same base compositions. A large fraction of the 27 eukaryotes exhibited significant over-representations (enrichment) of longer length (N ≥ 9 bp) poly(dA) and poly(dT) tracts in their intron and flanking sequences, but not their coding sequences. This occurred in a novel base composition dependent fashion. A much smaller number of the 27 organisms exhibited significant over-representations of longer length (N ≥ 9) poly(dG) and poly(dC) tracts. For *P. falciparum *and *S. cerevisiae *single gene containing sequences as well as whole chromosomes, all homopolymer tracts were found to have similar length dependent frequencies and therefore over-representation in their functional compartments.

## Results

The purpose of this study was to reveal similarities and differences in the frequency of occurrence of homopolymer tracts of varying lengths in different eukaryotic sequences across the biological range of base compositions from 20 – 60% (G+C). Primarily single gene containing sequences of 27 organisms were investigated. This was done on purpose since most of the organisms had largely only single gene containing sequences available in the public databases. Restricting our comparative analysis in this fashion ensured that the results for all organisms could be easily compared. A total of 25,109 sequence documents were collected. As we mentioned earlier, there are often a significant level of redundancies in the sequences found in the public databases. CleanUP is a program we used to remove those redundancies, and following its application, 5.2 × 10^7 ^bases from 15,560 cleaned sequence documents were analyzed and compared [36]. As shown in Table 1 comparing columns before and after application of CleanUP, there are varying levels of redundancies in some of the original collected files for different organisms in the public database. In Table 1, we list the complete name of each organism as well as an abbreviation that is used throughout the following discussion of our Results.

**Table 1:** Summary of the sequence files of the 27 organisms studied

**ORGANISM**	**ABBR.**	**DNA type**	**DOCUMENTS BEFORE CLEAN-UP**	**DOCUMENTS AFTER CLEAN-UP**	**TOTAL (bp)**	**TOTAL GC%**
Dictyostelium discoideum	Dd	single genes	492	440	966261	25.70
Plasmodium falciparum	Pf	single genes	1652	790	1065133	27.11
		chromosome II, III	2	2	2007209	19.82
Tetrahymena thermophila	Tt	single genes	109	97	214676	28.89
Candida albicans	Ca	single genes	439	378	959035	35.21
Manduca sexta	Ms	single genes	54	46	119657	35.62
Caenorhabditis elegans	Ce	single genes	234	221	1319875	36.92
Schizosaccharomyces pombe	Spo	single genes	813	720	1346439	37.54
Arabidopsis thaliana	At	single genes	1908	1520	3139637	38.09
Schistosoma mansoni	Sm	single genes	98	74	125605	38.37
Danio rerio	Dr	single genes	339	266	603534	38.60
Saccharomyces cerevisiae	Sc	single genes	2249	928	3815906	38.68
		chromosome I-XVI	16	16	11426263	38.45
Drosophila melanogaster	Dm	single genes	1883	968	3459297	39.88
Strongylocentrotus purpuratus	Spu	single genes	153	113	116647	41.78
Xenopus laevis	Xl	single genes	568	411	805354	41.99
Oryza sativa	Os	single genes	605	507	1514656	44.61
Trypanosoma brucei	Tb	single genes	457	368	1039208	46.17
Fugu rubripes	Fr	single genes	216	181	1737132	46.30
Zea mays	Zm	single genes	629	480	1225027	46.92
Mus musculus	Mm	single genes	9288	5179	11191148	47.36
Anopheles gambiae	Ag	single genes	78	43	73592	48.20
Gallus gallus	Gg	single genes	1868	1061	1910331	50.00
Toxoplasma gondii	Tg	single genes	195	117	284187	50.70
Emericella nidulans	En	single genes	72	62	165648	51.07
Aspergillus niger	An	single genes	215	160	386447	52.65
Neurospora crassa	Nc	single genes	252	217	494046	53.37
Leishmania major	Lm	single genes	89	81	129155	59.11
Chlamydomonas reinhardtii	Cr	single genes	136	114	349624	61.84

The base compositions of the total sequence population from each organism ranges from a low of 25.70% (G+C) for *D. discoideum *(Dd) to a high of 61.84% for *C. reinhardtii *(Cr). From the standpoint of (G+C)%, the organisms we investigated are not evenly distributed. If we take as the midpoint, *G. gallus *(Gg), whose (G+C)% is exactly 50%, there are only 6 organisms over 50%, while the rest (19 of 27) are all below 50%. This is due to the fact that there are more available sequenced eukaryotic organisms that are AT-rich than GC-rich. In this study, we dissected DNA sequences into coding, intron and flanking functional compartments as shown in Table 2. In every instance, the non-coding regions (intron and flanking) were found to be significantly more AT-rich than coding sequences.

**Table 2:** The (G+C)% in different compartments of the 27 organisms

**ORGANISM**	**ABBR.**	**FLANK (bp)**	**GC%**	**INTRON (bp)**	**GC%**	**CODING (bp)**	**GC%**
Dictyostelium discoideum	Dd	207722	15.26	22407	11.14	621748	30.32
Plasmodium falciparum	Pf	143401	16.27	18597	12.64	840733	29.41
Tetrahymena thermophila	Tt	60095	20.99	8624	20.14	101889	34.37
Candida albicans	Ca	302347	31.26	3035	33.05	631769	36.93
Manduca sexta	Ms	43331	33.38	17299	30.87	21979	47.79
Caenorhabditis elegans	Ce	737951	32.87	121316	33.07	384627	45.45
Schizosaccharomyces pombe	Spo	432364	32.67	22638	30.44	813101	40.41
Arabidopsis thaliana	At	1256492	33.20	270913	32.05	1015490	45.75
Schistosoma mansoni	Sm	37773	36.12	11760	35.94	39913	40.05
Danio rerio	Dr	232819	35.07	41465	34.21	147459	47.90
Saccharomyces cerevisiae	Sc	1124634	36.06	17273	32.90	2356815	40.14
Drosophila melanogaster	Dm	1238086	37.56	227141	39.54	747774	52.85
Strongylocentrotus purpuratus	Spu	38732	36.85	11450	34.43	24405	53.22
Xenopus laevis	Xl	266034	39.32	183822	38.36	164675	48.08
Oryza sativa	Os	567768	40.35	133898	36.74	394283	55.89
Trypanosoma brucei	Tb	120949	43.19	1621	44.97	415684	50.73
Fugu rubripes	Fr	624550	43.85	230616	43.22	342435	54.13
Zea mays	Zm	611822	43.30	114505	41.21	305091	55.88
Mus musculus	Mm	4571933	46.05	1708894	46.75	1475920	53.15
Anopheles gambiae	Ag	29527	42.93	8028	44.33	24151	56.93
Gallus gallus	Gg	563319	50.08	457259	46.15	369315	55.13
Toxoplasma gondii	Tg	100004	50.01	32167	49.49	94260	54.51
Emericella nidulans	En	75257	48.72	2888	47.13	83665	53.12
Aspergillus niger	An	136216	48.46	15832	45.95	188982	56.48
Neurospora crassa	Nc	154422	50.44	18685	48.88	254177	56.07
Leishmania major	Lm	34029	57.27	54	50.00	70014	60.71
Chlamydomonas reinhardtii	Cr	163614	59.70	49607	62.03	106939	66.55

### Long homopolymer tracts are over-represented in non-coding sequences of AT-rich eukaryotes

**Figure 1:**
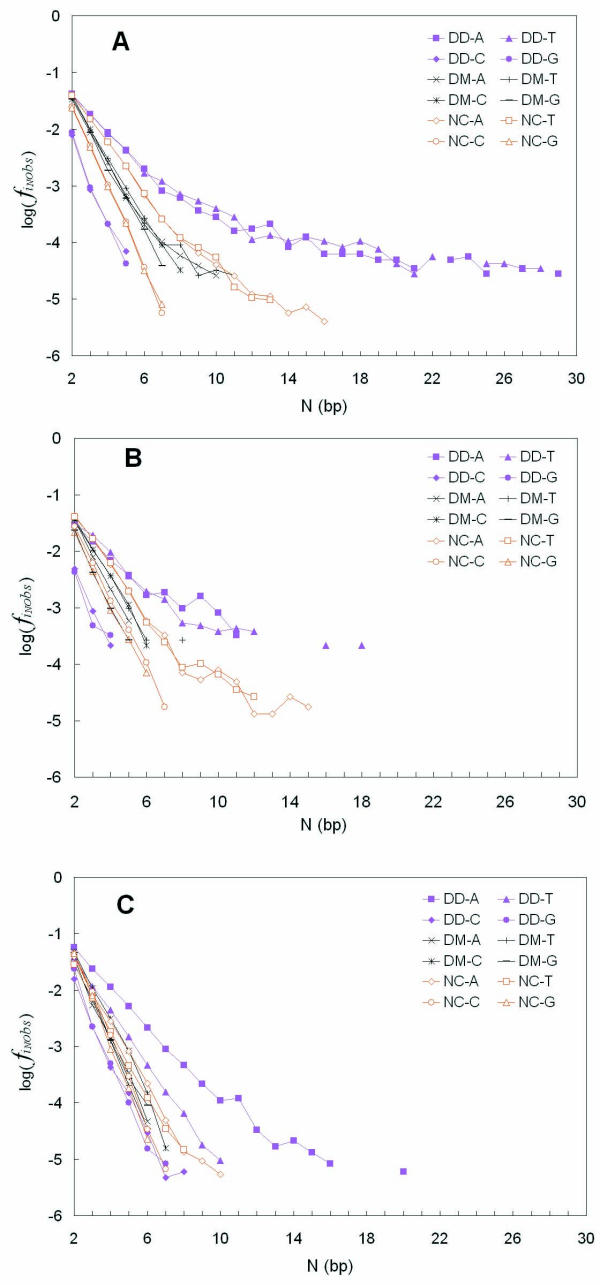
**Comparison of the observed length N dependent poly(dA), poly(dT), poly(dC) and poly(dG) tract frequencies found in sequences from different DNA functional regions from the organisms Pf, Dm and Nc **A. flanking sequences; B. intron sequences; C. coding sequences

The observed frequencies of non-overlapping base *i *tracts of length N, , in different DNA regions were analyzed as a function of tract length N in all 27 organisms. In Figure 1, we present the results of analyzing all 4 base tracts from Pf, Dm and Nc sequences as representative examples. The total (G+C)% of the DNA analysed from these organisms is 27.11%, 39.88% and 53.37%, respectively, representing typical low, median and high base composition eukaryotes. Very long tracts (high N) are rare, leading to low counts and large fluctuations in log (). Therefore, for each organism, any tract count observed to be less than 4 for a given tract length N was excluded from the data analysis in order to eliminate noise in the data. Getting rid of the interference caused by noisy data enhanced and clarified the comparisons we made from slope determinations. Some of the points in Figure 1 are not connected because they did not present contiguous data along the x-axis (tract length N).

From Figure 1A, it is clear that the frequency of poly(dA).poly(dT) tracts in the very AT-rich Pf flanking regions are much higher than Dm and Nc tract frequencies. This becomes more pronounced when the tract length N becomes larger (N ≥ 7 bp) and reaches a maximum at around N ≥ 10 bp. On the other hand, the frequencies of poly(dG) or poly(dC) tracts in Pf are significantly lower and no differential N dependence is observed. Meanwhile, no significant difference can be observed between Dm and Nc, except for the higher  values at longer N in Nc sequences. Similar behavior is also observed in intron sequences in Figure 1B. The over-representation of poly(dA) or poly(dT) tracts in intron regions is also evident at higher N values. However, this behavior is almost non-existent in coding regions (Figure 1C), except for Pf, which is the most AT-rich eukaryote in all the 27 of our survey. This organism exhibited over-representation of poly(dA) tracts in its coding region as well as poly(dT) tracts as we shall see below.

It is also very clear that the longer poly(dA) and poly(dT) tracts, usually of length larger than 20 bp, are only detected in flanking regions. In all cases, the plotted curves exhibited a transition region of changing slope, points falling between tract lengths 6 bp and 9 bp. This behavior, as we have described previously in *D. discoideum *DNA, leads one to conclude that the long poly(dA) and poly(dT) tracts are over-represented relative to random tract occurrence in random DNA sequences of equivalent base composition [1]. This is a fact that we illustrate and quantitate later in this study. By contrast, the nearly linear relationship of points in Figure 1, for all the organisms' tracts of all types at lengths N ≤ 6 bp indicates a similarity that differs for each organism only by the individual linear relationships being offset from each other. This is a trivial consequence of the different base compositions of the DNAs, giving rise to frequencies of any given tract at levels near those expected based on random occurrence in that base composition.

### Comparing tract frequencies from single genes with those from whole chromosomes

**Figure 2:**
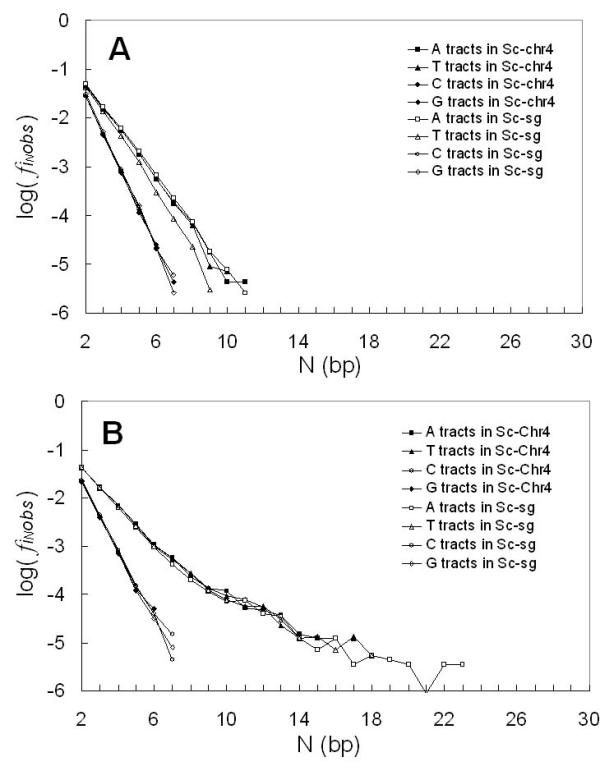
**Comparison of the four tract frequencies from Sc chromosome 4 sequences and Sc single gene sequences as a function of N, the tract length, calculated from: **A. coding sequences; B. non-coding sequences. In the legend, "sg" represents "single gene" and "chr" represents "chromosome".

In order to confirm that there is consistency in the homopolymer tract frequency levels between single gene data and whole chromosome data in any given organism, we collected single gene data and whole chromosome data separately for representative organisms – Pf and Sc, where fully annotated whole chromosome sequences were available. For Pf, we collected chromosomes II and III. For Sc, we collected sequences for all 16 chromosomes. The single gene data were compared to the chromosome data of each organism respectively. For both organisms, the comparison results are similar and, therefore, we only display in Figure 2 representative data here for Sc single gene data compared with Sc chromosome IV, the largest of the 16 chromosomes. Since the whole chromosome data is only annotated with coding and non-coding regions, we combined the results from single gene data for intron and flanking regions, which were previously separated in our Figure 1 analysis, in order to make a consistent comparison with the whole chromosome data. Aside from poly(dT) tracts in coding sequences, the analyses showed no consistent significant differences. Therefore, we judge that our conclusions using single gene data are representative of whole chromosome data for the 27 eukaryotes we analyzed in this survey.

### Quantitating the over-representation of tracts

**Figure 3:**
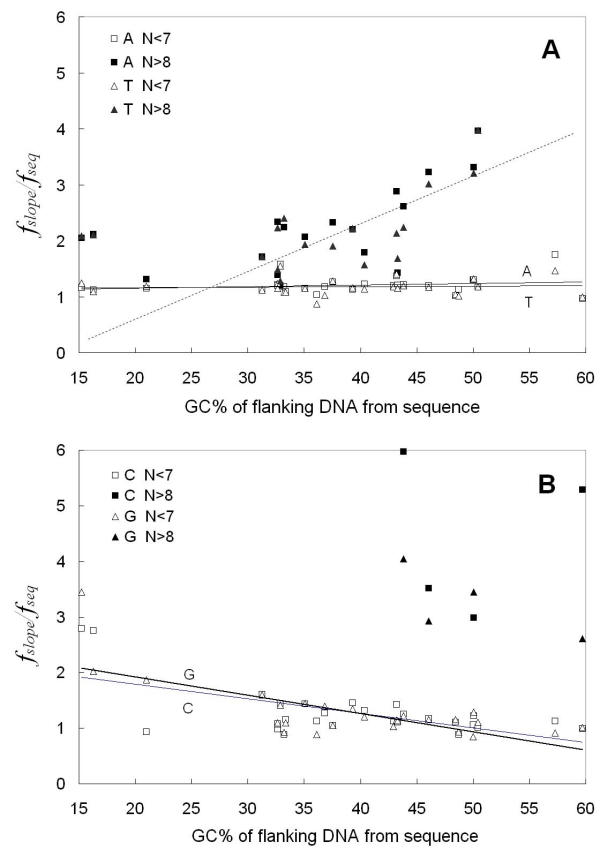
**Comparison of the frequency ratio, fslope/fseq, to the real (G+C)% of the particular organisms' flanking DNA. The fslope is calculated from the slopes of Figure 1 types of graphs for short (N ≤ 6 bp) and long (N ≥ 9 bp) tract data found in flanking sequences from 27 organisms. **A. poly(dA).poly(dT) tracts. The straight solid lines are linear regression fits for short tracts (N ≤ 6 bp) of each type and the dashed line (R^2 ^= 0.5591) demonstrates the trend in long (N ≥ 9 bp) tracts; B. poly(dG).poly(dC) tracts. The straight lines are linear regression fits for short tracts of each type (N ≤ 6 bp).

**Figure 4:**
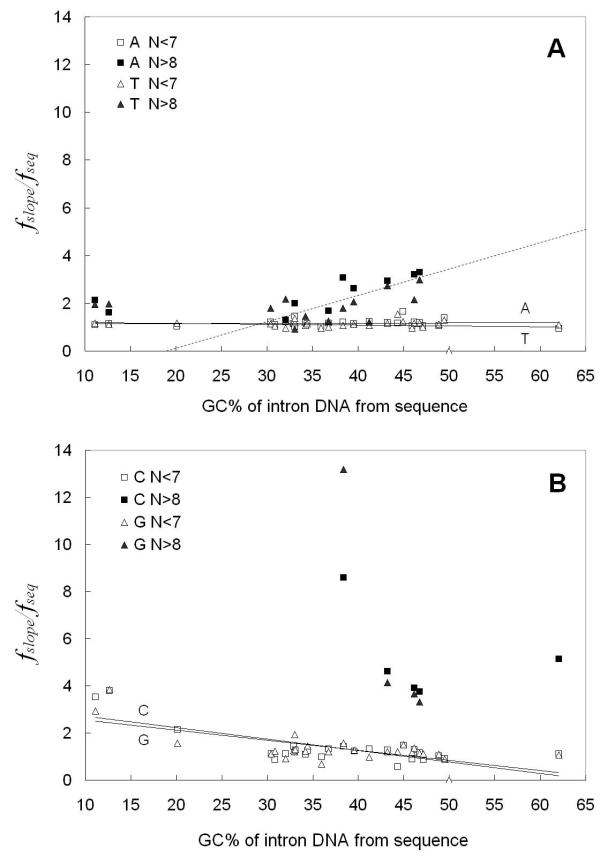
**Comparison of the frequency ratio, *f*_slope_/*f*_seq_, to the real (G+C)% of the particular organisms' intron DNA. The fslope is calculated from the slopes of Figure 1 types of graphs for short (N ≤ 6 bp) and long (N ≥ 9 bp) tract data found in intron sequences from 27 organisms. **A. poly(dA).poly(dT) tracts. The straight lines are linear regression fits for short tracts (N ≤ 6 bp) of each type and the dashed line (R^2 ^= 0.5474) demonstrates the trend in long (N ≥ 9 bp) tracts; B. poly(dG).poly(dC) tracts. The straight lines are linear regression fits for short tracts of each type (N ≤ 6 bp).

**Figure 5:**
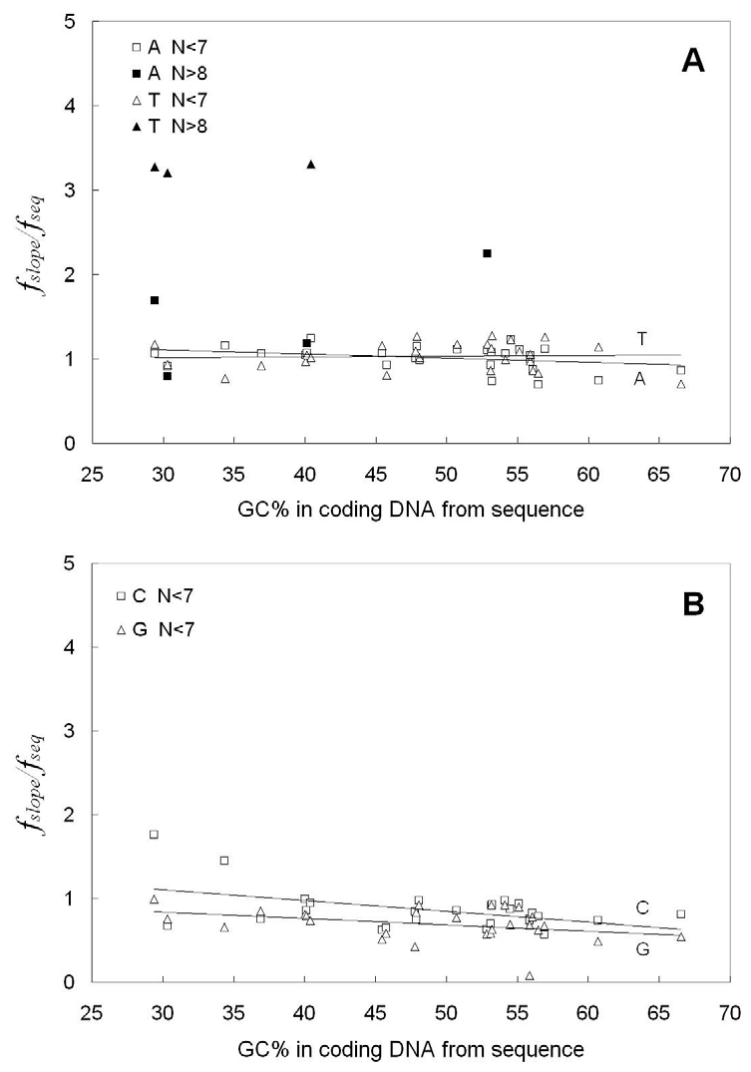
**Comparison of the frequency ratio, fslope/fseq to the real (G+C)% of the particular organisms' coding DNA. The fslope is calculated from the slopes of Figure 1 types of graphs for short (N ≤ 6 bp) and long (N ≥ 9 bp) tract data found in coding sequences from 27 organisms**, A. poly(dA).poly(dT) tracts. The straight lines are linear regression fits for short tracts (N ≤ 6 bp) of each type; B. poly(dG).poly(dC) tracts. The straight lines are linear regression fits for short tracts (N ≤ 6 bp) of each type.

We next wished to quantitatively compare for all 27 eukaryotes, the differences between the length N dependent frequencies of short tracts (N ≤ 6 bp) and long tracts (N ≥ 9 bp) of the type that we presented in Figure 1. We designed the data analysis method by separating the Figure 1 x-axis into two regions of different tract behavior: N ≤ 6 bp and N ≥ 9 bp. The data points in the short tract range and those in the long tract range were treated separately. For short and long tract point regions separately, the average frequency, *f*_*slope*_, of the tract base *i *in the particular genome compartment were calculated. The *f*_*slope *_parameter is the "effective" base *i *frequency for the sequences in that DNA compartment that would give rise to the observed log () vs. N dependent tracts frequency behavior in that region based upon the eqn. [1b] random model. The *f*_*slope *_is obtained from the inverse of P', where the slope [-log(P')] is obtained from the log () vs. N type plots in Figure 1, fit by eqn. [1b], as we present in Methods and have previously described [1]. This is a model that assumes random occurrence of tracts. Although it is known that DNA sequences do not occur randomly and that 1^st ^Order Markov chain behavior can describe some of the behavior of eukaryotic sequences, we have chosen here to compare the occurrence of tracts in real sequences to that of tracts in random DNA of equivalent base composition because the comparison is intuitively easy to grasp. The results from all 27 organisms are presented here in Figures 3, 4 and 5 for flanking, intron and coding sequences respectively. The data are plotted as frequency ratios (*f*_*slope*_/*f*_*seq*_: where *f*_*seq *_is the actual base frequency tabulated from all bases comprising the sequences in the real sequence compartment) plotted versus the overall real (G+C)% of each individual DNA sequence compartment studied. The higher the frequency ratio in Figures 3, 4 and 5, the higher is the enrichment or over-representation of the tract. It is a common feature for all the organisms that when N ≤ 6 bp, the ratio is near 1. Therefore, in all the Figures 3,4,5, the trend lines developed by the linear regression fit of only the N ≤ 6 bp data have slopes close to zero and intersect the y-axis at a ratio near 1 to 2. The regression lines extrapolating to a ratio near 1.0 (Figure 3A, Figure 4A and Figure 5A) indicate that N ≤ 6 bp tracts occur at frequencies expected for the base compositions found in each of the organisms' sequence compartments. Interestingly, this behavior occurs for the poly(dA).poly(dT) tracts in all three functional compartments – coding, intron and flanking DNAs. On the other hand, for N ≤ 6 bp poly(dG).poly(dC) tracts of all organisms, the regression lines all have a slightly negative slope, with flanking and intron sequences (Figure 3B and Figure 4B, respectively) exhibiting an intercept ratio of 2 or greater. This clearly indicates a trend to greater over-representation of short poly(dG).poly(dC) tracts in organisms of higher (A+T)% base composition.

For N ≥ 9 bp tracts in coding sequences (Figure 5A &5B), there were not enough tracts to allow calculation of *f*_*slope *_values from the Figure 1 type data. However, in flanking and intron sequences (Figure 3A and Figure 4A), the ratio was determined and is much higher than 1 for all the organisms, indicating that the poly(dA).poly(dT) tracts are significantly over-represented. The behavior of poly(dA).poly(dT) tracts in both flanking and intron sequences are similar and demonstrate a novel and interesting dependence of over-representation on the base composition of the organism's DNA. Starting at 30 %(G+C) and increasing to 50 %(G+C) (note linear fit trend line), these tracts are increasingly over-represented as the ratio trends from around 1.5 up to 4.0.

For lengths N ≥ 9 bp in Figures 3B and 4B, we observed similar behavior for poly(dG).poly(dC) tracts. In a few organisms between 35%–50% (G+C), there is a high ratio of frequencies indicating a high level of over-representation. However, longer poly(dG).poly(dC) tracts are not over-represented and do not occur at long lengths as we show later in nearly as many organisms as we observed for poly(dA).poly(dT) tracts. Therefore, even though there appears to be evidence for a trend in these figures, we have not indicated with a negative slope linear trend line, mirror-image trend behavior to that exhibited by the poly(dA).poly(dT) tracts.

### The over-representation of long poly(dA).poly(dT) tracts exhibit exponential frequency increases compared to predicted values

**Figure 6:**
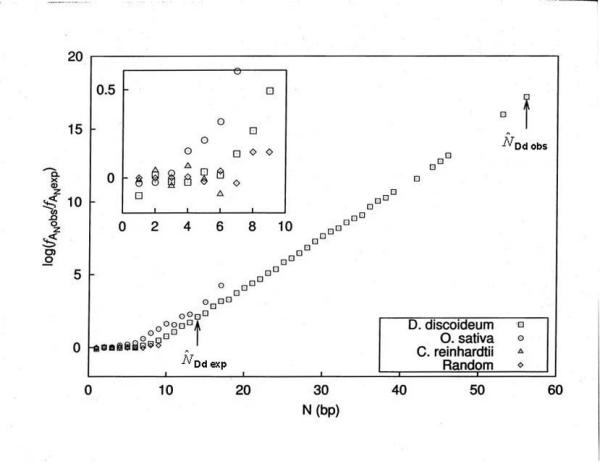
**Comparison of *f*_obs _*vs*. *f*_exp _calculated for eukaryotes Dd, Os, Cr and a randomly generated sequence as a function of the tract length N. The ****and ****values for *D. discoideum *are presented **Due to the small differences exhibited between the organisms when N is small, we present the small inset figure for the region from N = 0–10 bp enlarged for clarity.

In order to show more clearly the genomic over-representation of the long poly(dA) and poly(dT) tracts, we introduced a variable, , representing the predicted frequency of base *i *at length N based on random tract occurrence in DNA of equivalent base composition. Eqn. [2] is used to calculate . The ratio of  equals *R*, the Threshold. In Figure 6, we plot log *R *vs. N for only the poly(dA) tract data from Dd, Os, Cr, representing genomes of low, median and high (G+C)% base composition, respectively. Similar comparative data for all 27 organisms was determined but is not shown here. For comparison purposes, we include in Figure 6 the tract frequency results determined for a random sequence of 10^6 ^nucleotides of 50% (G+C) composition generated with a random number generator. Each base position in that random sequence was picked from all 4 bases having equal probability (0.25) of being selected. In the randomly generated sequence, there were no tracts longer than 9 bp. The small inset panel in the upper left corner of the figure presents an enlarged view of N from 0 to 10. In this panel, as expected, the random sequence exhibits points with values closely centered around 0 on the y-axis. The only exceptions are for the points N = 6 and higher that are noisy and exhibit fluctuations as high as 0.15, due to the low number of tracts occurring at those sizes. Thus, there are no significant differences between  and  and . In contrast, the results from real organisms show very different behaviors. There are two regions, a linear part with slope around 0 when N is relatively small and an exponentially increasing frequency ratio when N increases beyond a certain value. In Figure 6, two of the three organisms exhibit this exponentially increasing ratio. Os is the first to go above the 0 line when N is around 3 bp. A similar change to an exponentially increasing ratio was observed for Dd at a different N value of about 7 bp. This is the same tract size where we previously observed that poly(dA) and poly(dT) tracts begin to exhibit over-representation [1]. Of all the organisms, the Dd data exhibited one of the most significant over-representation levels- a 10^13^-fold enrichment of these tracts occurring at lengths about 45 bp.

Another significant feature of the Dd data, is the large difference between  and . This indicates that a high over-representation of long poly(dA).poly(dT) tracts likely occurs in this organism, as we saw in Figure 2. It also makes clear that this organism utilizes poly(dA).poly(dT) tracts to sizes at least 40 bp longer in length than would be expected,  = 14 bp, based upon the random tract occurrence calculated from its base composition. It is also the highest over-proportional tract size we observed, as we present later.

### Poly(dA).poly(dT) tracts show inverse correlation between (G+C)% composition and threshold value

**Figure 7:**
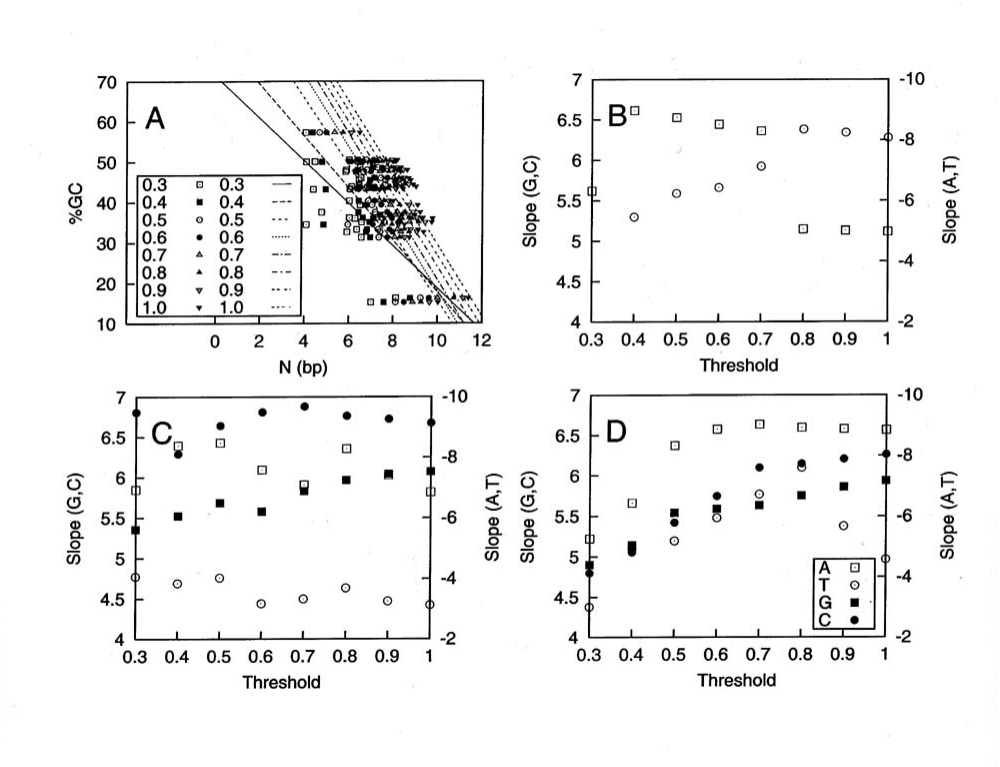
**The (G+C)% dependence of a series of calculated threshold values for enrichment of each homopolymer tract type **In panel A. data is presented for the length N observed at the given series of threshold values for poly(dA) tracts from all 27 organisms. Slopes determined for each threshold from the type of representative data presented in A. were then calculated from all 27 organism to provide the values for poly(dG).poly(dC) and poly(dA).poly(dT) tracts within: B. coding; C. intron; D. flanking regions. The legend in panel D applies as well to panels B and C.

The concept of a threshold value was introduced to provide a description of the N dependence of the observed frequency of the tracts, , as it begins to rise significantly above that of the calculated . The threshold is a particular value of the log(*R*), where *R *is defined by eqn.3. In this study, we chose not to attempt a universal definition of the over-representation criterion. Rather, we decided to examine various thresholds that defined different over-representations beginning at 0.3, where the  is 2 times greater than the , with increasing values up to 1.0, where the  is exactly 10 times that of the . We present these data in Figure 7A for poly(dA) tracts, where the N values achieved at the different thresholds are plotted *vs*. the (G+C)% composition of the DNA. A negative slope between (G+C)% and N at threshold for poly(dA) tracts in flanking sequences was observed for all thresholds. Poly(dT) tracts displayed similar behavior (data not shown). The changing slopes of the linear correlation lines shown in Figure 7A exhibit a progression from highest negative slope at threshold 0.3 to lower negative slope at threshold of 1.0. Thus, the lower the (G+C)% base composition of the genome, the higher the N at which over-representation of poly(dA) tracts occurs. For poly(dC) and poly(dG) tracts in flanking sequences, a positive linear correlation between the (G+C)% base composition and N at threshold was observed (data not shown). Interestingly, the slopes of the correlation lines also resulted in a progression of slope values. Thus, for poly(dC) tracts in flanking sequences, the lower the genome (G+C)% base composition, the lower the N at which over-representation occurs.

For homopolymer tracts of each type in coding, intron and flanking DNAs, data of the type shown for poly(dA) tracts in Figure 7A were calculated and the linear fit slopes are presented in Figure 7 panels B-D, respectively. For all the data, the poly(dA).poly(dT) tracts exhibit uniformly negative slopes between -4 and -10, while the poly(dG).poly(dC) tracts all exhibit positive slopes between 4.5 and 7. In coding sequences, poly(dA) tracts showed a sharp drop between 0.7 and 0.8 while poly(dT) tracts exhibited a slow increase. No poly(dG) or poly(dC) tracts of significant length occur in coding DNA, which did not allow over-representation to be exhibited at these threshold values. Therefore, no slope points are shown. For intron sequences in panel C, poly(dG).poly(dC) tracts exhibited no significant consistent slope trend. However, poly(dC) tracts have overall greater slopes than poly(dG) tracts. Likewise for poly(dA).poly(dT) tracts, no trend is evident but the latter possesses significantly greater slopes than the former. For flanking sequences, all four tract types exhibited increasing slopes as threshold values increased. As was true for intron sequences, poly(dC) again had overall somewhat greater slopes than poly(dG) tracts. Similar behavior was observed for poly(dA).poly(dT) tracts in both intron and flanking DNA sequence types, with poly(dA) again occurring at greater slopes than poly(dT).

### The highest over-representation and over-proportional length of homopolymer tracts appear in median GC% organisms

**Figure 8:**
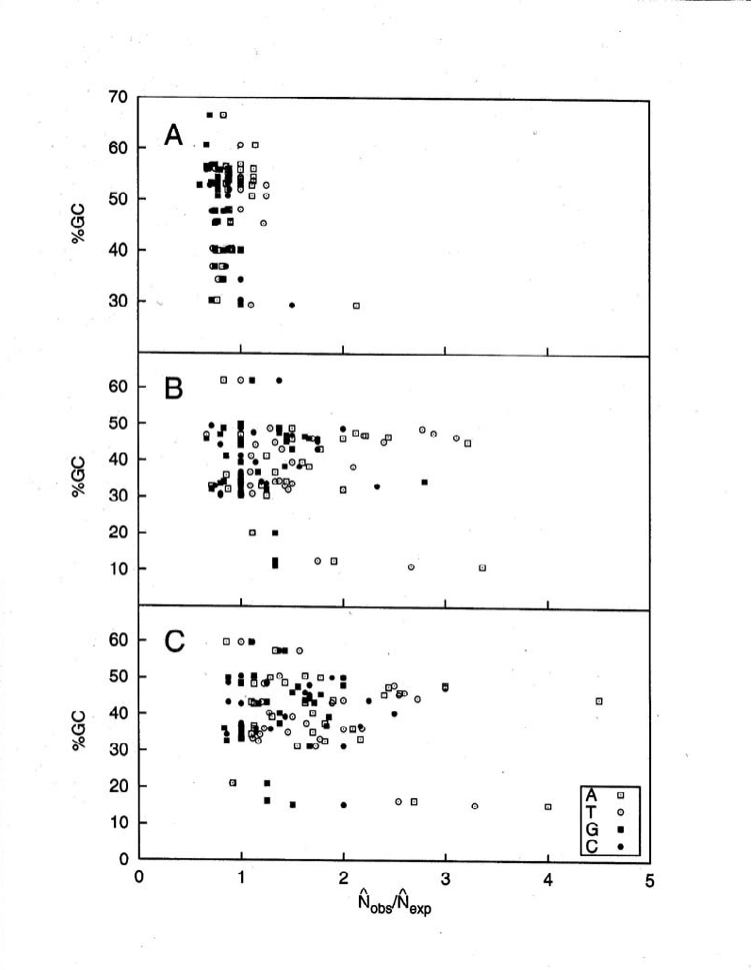
**The relationship of the (G+C)% of the DNA analyzed to the calculated ** / **(P) for all the sequences of 27 organisms **A. coding; B. intron; C. flanking.

We next used the proportion, *P*, eqn.5 for all the 4 homopolymer tracts to compare the maximum observed tract size with the maximum tract size expected for random tract occurrence within that (G+C)% base composition DNA. If the *P *quantity is greater than 1, tracts are over-proportional in length and if *P *is less than 1, tracts are under-proportional in length. We present *P*,  / , for coding, intron and flanking DNAs from all 27 organisms in Figure 8 panels A-C, respectively. The  values are calculated from eqn.4. Large differences are obvious between coding and non-coding sequences. It is clear that tracts in coding regions, being mostly less than 1, are under-proportional in length for all base types. However, poly(dA).poly(dT) tracts are slightly under-proportional in length in GC-rich organisms, a fact that agrees with our previous observation of over-representation in tract frequencies in Figure 5.

By contrast, the average behavior of intron and flanking regions in Figure 8 is that tracts of all types, but especially poly(dA).poly(dT) tracts, occur at significantly over-proportional lengths. This fact is consistent with their significant over-representation levels that we previously presented in Figures 3,4,5. Very long poly(dA).poly(dT) tracts are observed in non-coding regions of some organisms, at lengths greater than 20 bp in excess of the expected length. Interestingly, the highest over-representation levels of tracts are found in organisms between 30% – 50 % (G+C) base composition. The only exception to this was found in Dd, the most AT-rich organism we studied, where the longest poly(dA) tracts were 71 bp. Higher poly(dG).poly(dC) tract frequencies than expected for organisms greater than 40 % (G+C) base composition were observed in Figure 3B and Figure 4B. The same was true for the most AT-rich ones – Dd and Pf. Figure 8 panels B and C correspondingly exhibit significant levels of over-proportional lengths of poly(dG).poly(dC) tracts, consistent with over-representation, for organisms greater than 30% (G+C) base composition and exhibit moderate over-representation of poly(dG).poly(dC) tracts for Dd and Pf.

## Discussion

As a result of recent progress in the rate of DNA sequencing, the amount of sequenced DNA from many organisms has grown significantly. This has allowed our systematic study of the behavior of non-overlapping homopolymer tract frequencies in the 27 eukaryotes in this study spanning the 20% – 60 % (G+C) base composition range. Pre-processing of each of the 27 eukaryotes' largely single gene containing sequence files eliminated sequence redundancies that would introduce bias into the frequency calculations [39-41], that would not be representative of the biological genomes. In most organisms, well over 10% of the documents obtained were judged to contain redundant sequences and were removed by the CleanUP program (Table 1).

From our results in this study, it is clear that long homopolymer tracts are over-represented in non-coding sequences, but not coding sequences, within eukaryotic genomes of all base compositions. This is perhaps not surprising considering that the coding sequence populations must satisfy the constraints of the triplet genetic code. In addition, organisms might minimize the numbers of tracts in coding regions to avoid the severe, even fatal frame-shift mutations that might be introduced by slippage-replication events at tracts [3-5,42]. In nearly all the organisms we studied, poly(dA).poly(dT) tracts were very much over-represented, beginning to be significantly enriched at lengths around 4–10 bp. These tracts also occurred at over-proportional lengths. This was particularly the case for organisms between 30% – 50% (G+C) composition, where over-proportional lengths were pronounced. By contrast, poly(dG).poly(dC) tracts, somewhat over-represented, do not occur at over-proportional lengths. This extends the findings of our previous *D. discoideum *DNA study that first described the tract over-representation transition region occurring at around 8–10 bp for poly(dA).poly(dT) sequences and their high over-proportional lengths [1]. Somewhat similar observations were made in a subsequent study of five eukaryotic organisms [35]. In general studies of repetitive sequences, poly(dA).poly(dT) tracts have been observed to be over-represented within eukaryotic genomes while poly(dG).poly(dC) tracts are significantly rarer [2,43]. Specific human repetitive sequences, such as the Alu elements, have been shown to contain long poly(dA).poly(dT) tracts, representing a significant repetitive sequence location for some of the over-represented tracts we observed in this study [44].

It has been suggested in a previous study that the over-representation occurring around 7–10 bp represented the minimum thermodynamic length required for any simple sequence repeat such as homopolymer tracts to undergo expansion by slip strand replication [35]. However, in our current study of 27 eukaryotes of widely varying base composition, we present more extensive results, especially those in Figure 7, that demonstrate this is not the case. Depending upon the threshold tract size value chosen to express over-representation of the tracts, the N value where over-representation occurs for A tracts can be seen in Figure 7A to range for all the organisms from as low as 4–6 bp for 0.3 threshold (2× enrichment) to 8–11 bp for 1.0 threshold (10× enrichment). Furthermore, for poly(dA).poly(dT) tracts, the (G+C)% base composition *vs*. N slopes are negative for all thresholds, while for poly(dG).poly(dC) tracts the slopes are positive for all thresholds. This means that the base composition of the organism is the most important determinant of the particular threshold N value where over-representation begins and argues against an absolute solely thermodynamic determinant to the N value where over-representation begins *via *slip strand replication. In fact, our observed negative slopes for the poly(dA) tracts in Figure 7A, means that in higher (G+C)% composition organisms, the poly(dA) tracts become enriched at shorter N values than in (G+C)% poor organisms. This result is counter-intuitive to a thermodynamic argument, since the high (G+C)% base composition in neighboring sequences around a short poly(dA) tract in a high (G+C)% organism would be expected to resist the tract looping out to allow for slip strand replication because of the higher level of base stacking stabilization energy in the (G+C)-rich neighboring sequences. We believe that these (G+C)% composition dependent variable threshold N values we observed here are describing a complex mechanism that determines successful tract lengthening, rather than a single thermodynamic criterion for successful DNA looping during slip strand replication.

The reason why poly(dG).poly(dC) tracts occur only at short lengths in eukaryotes may have to do with some interesting structural and energetic polymorphisms of these sequences. Even short tracts of this type have the ability to rearrange from the right-handed double helix to form G-quadraplex structures. These structures have been implicated in biological function in systems as diverse as eukaryotic immunoglobulin switch regions [45], telomeric repeats on chromosome ends [46] and promoter regions [34]. Therefore, eukaryotes may select against these tracts at any significant length in order to minimize problems resulting from the significant structural plasticity of these tracts. Another potential problem with these tracts is the fact that they represent potential reservoirs of oxidative damage. Recently, long-range electron transfer has been demonstrated to occur through the delocalized molecular orbitals of the stacked bases in the DNA double helix [47]. The electron transfer energy in these studies is insensitive to distance along the helix, but is sensitive to the level of base stacking. Therefore, these electron transfer events ultimately cause oxidative damage at GG dinucleotides, a base pair doublet that has high stacking levels. Even greater intensities of photo-damage were observed for GGG triplets. Therefore, eukaryotic organisms have a second compelling reason to mostly avoid the use of these homopolymer tracts at any significant length-a fact reflected in the data we have presented here.

It must be kept in mind in these discussions of homopolymer tract over-representation, that these tracts represent only a subset of the larger sequence class of polypurines and polypyrimidines that exist in and are over-represented within all eukaryotes. In a study of over 700 sequenced chromosomes or long sequences contained in plasmids [48], a bias toward longer polypurine and polypyrimidine tracts in eukaryotes was reported as a function of length N, similar to the homopolymer poly(dA).poly(dT) tract frequency behavior we have reported here.

We have previously observed that the long (N>10 bp) poly(dA).poly(dT) tracts over-represented in *D. discoideum *DNA (1) were not randomly distributed within the sequences from that organism. In fact, they are arrayed with an average spacing that corresponds to the repeating nucleosome DNA length found experimentally in *D. discoideum *chromatin [18]. And in that study, adjacent long pairs of tracts plus the intervening non-tract DNA were found to occur within a length corresponding to the internucleosomal linker DNA size found in *D. discoideum *chromatin. These results suggest that the long tracts only occur in restricted locations in chromatin. This supposition is supported by more recent experimental studies in *D. discoideum *chromatin compared to calculations of poly(dA).poly(dT) tract spacings in *D. discoideum *DNA (Marx, K.A., Zhou, Y. and Kishawi, I. unpublished results). That long poly(dA).poly(dT) tracts avoid being located within nucleosome core regions was experimentally determined from sequencing studies of native chicken erythrocyte chromatin [17]. In agreement with this line of reasoning, recent studies have shown that the nucleosome structure readily incorporates DNA containing short tracts, such as the sequence A_5_TATA_4_, but longer tracts such as those found in the sequence A_15_TATA_16_, completely disrupt the phasing of nucleosomes [49]. Short tracts not only are incorporated into nucleosomes, but they actually represent more stable than average nucleosome positioning sequences when they occur in-phase with the helical turn at roughly every 10 bp [50]. In human NF1-the Alu repeat element is blocked *in vitro *from forming a nucleosome by the presence of a bipartite T_14_A_11 _tract sequence [51].

Different investigators have postulated two additional functions for tracts. The first is their use as promoters. This function may be synergistic with the long poly(dA).poly(dT) tracts preventing the formation of nucleosome structures. The second is as DNA binding sequences for specific poly(dA).poly(dT) tract binding proteins that possess some as yet unknown function. There are a number of reports that poly(dA).poly(dT) tracts function in eukaryotes as promoter sequences. In *D. discoideum *DNA, the actin genes contain a remarkably long (45 bp) promoter upstream of the TATA box [52]. In this study, the length of the tract was shown to correlate with the transcriptional level of these genes. A number of studies have demonstrated similar long tract promoter activity in yeast promoter regions [32,53,54] and in various mammalian [55] and human promoters [56]. In many of these studies, it was demonstrated that the promoter activity of the long tracts was correlated with this sequence being nucleosome free or not complexed with a protein.

In the case of potential tract function where proteins bind to long poly(dA).poly(dT) tracts, there are a few investigated examples. The small protein datin, 13 kD, has been isolated from *S. cerevisiae *cells [57]. It has a required tract-binding site that is 9–11 bp in length and its function upon tract binding is unknown. Two high affinity poly(dA).poly(dT) tract-binding proteins, 70 and 74 kD species of unknown function, have been identified in *D. discoideum *[58]. Another example of a tract binding protein has been discovered in *D. discoideum*, where some 200 copies of terminal repeat retrotransposons are under transcriptional control by a 134 bp DNA control element [59]. Within this control element, a nuclear protein called CMBF binds to two almost homopolymeric 24 bp poly(dA).poly(dT) sequences. This CMBF protein contains so-called 'A.T hook' regions that interact with a 5–6 contiguous A:T base pair tract. These 'A.T hooks' are found in a number of other (A+T)-rich sequence binding proteins such as HMG-I, DAT1 from yeast, D1 from *D. melanogaster *and human UBF. In summary, it is unclear what functions these various pure poly(dA).poly(dT) tract binding proteins serve, and how their binding occurs at specific tracts while other tracts remain free of protein. The one point that can be stated with certainty is the correlation between tract binding site size (8–11 bp) of the proteins and the upper threshold (8–11 bp) size where tracts become significantly over-represented or enriched in our study. We believe that this similarity is not coincidental and is a consequence of some functional linkage.

A novel aspect of our study was that for both flanking and intron sequences the over-representation of the poly(dA) and poly(dT) tracts were actually more pronounced in less (A+T)-rich organisms as compared to the most (A+T)-rich, Dd and Pf. Also novel was that poly(dA) and poly(dT) tracts showed negative slopes between the organisms' DNA (G+C)% composition and the threshold value. Thus, the higher the (G+C) base composition, the lower the tract length at which over-representation occurs. In fact, the highest over-representations of homopolymer tracts were observed in median (G+C)% organisms from 30–50%. Also, the distribution was almost symmetric with respect to the different organisms' (G+C)%. We believe that these results could be explained as a result of the insertion of retrotransposon elements into DNA. Eukaryotic transposons are known to be a widely occurring class of repeated DNA sequences ranging in size from about 1 kb to 8 kb. They contain inverted sequence repeats at their termini. The most common transposon class is comprised of retrovirus-like transposons [60], thought to arise from the integration of retroviral RNA sequences into a given eukaryotic genome. The resulting retrotransposon elements do not represent infectious viral DNA and are not transcribed since they lack accompanying promoter sequences. These DNA sequences do possess a poly(dA).poly(dT) tract that resulted from the 3' poly A tail on the original viral mRNA that formed the retrotransposon. Typical retrovirus-like retrotransposons, such as *copia *in *D. melanogaster *and *IAP *in *M. musculus*, are known to occur in thousands of copies in their respective genomes [60]. Therefore, inserted retrotransposon elements represent the likely origin of the excess over-representation of poly(dA).poly(dT) sequences that we observed in the majority of eukaryotes in this study, irrespective of their overall base composition.

## Methods

The single copy gene DNA sequences from 27 eukaryotic organisms were retrieved from GenBank, EMBL, and DDBJ, the members of the tripartite, international collaboration of sequence databases [61]. Every search excluded: ESTs (expressed sequence tag), STSs (sequence-tagged sites), and GSSs (genomic survey sequence), and were limited to organism and genomic DNA only. Moreover, sequences designated: "mitochondrion", "chloroplast", and "chromosome" were also excluded in the search query via these keywords using Boolean operators. In addition, the whole chromosome sequences from 2 of these organisms were also retrieved. The eukaryotic organisms covered are tabulated in Table 1.

The GenBank documents were processed by the program "CleanUP", kindly provided by the Department of Biochemistry and Molecular Biology, University of Bari, Italy [62]. Our purpose in using the program was to get rid of redundancy in our sequence collections so that no bias would be introduced into the homopolymer tract distributions we calculated [36]. The settings for the program are: precision factor (0), different adjacent nucleotides (2), threshold similarity percentage for searching (95.0), overlapping percent for searching (50.0), local similarity percent (70.0), percent ambiguous symbols (e.g. N) to skip matches (10), overlapping percent for cleaning (90.0), minimum length for overlapping (1), minimum length for overlapping segment (20), sequence minimum length so that a sequence is processed, otherwise is cleaned (30). This application of "CleanUP" results in eliminating all the sequences less than 30 bp in length, with more than 20 bp overlapping with the primary sequence (the sequence used use as a basis for comparison), and possessing over 90% similarity with the primary sequence.

Then the redundancy cleaned sequence files were input into the "Compile" program. Compile is part of the "MeltSim" program for the Windows suite of applications [37,63]. This program was used to extract raw sequences from the GenBank-formatted documents. Sequences of the functional categories, coding, intron and flanking were extracted according to their location tags. Respectively, "CDS" is for coding sequences, "intron" is for intron sequences, and "5'UTR", "3'UTR" and any other sequences excluding "CDS" and "intron" are all included into flanking sequences. The sequences were then concatenated into ASCII text files, one each for coding, intron and flanking. The ends of the individual sequences, as they appeared in the individual GenBank-formatted documents, were tagged to prevent the artifactual joining of those sequences that could result in the creation of artifactual long tracts. The basic characteristics of the coding, intron and flanking files that we used as computational start points for homopolymer tract frequency determination are summarized in Table 2.

Each file was subsequently analyzed using the program "Poly" [38,64], which calculates parameters for non-overlapping homopolymer tracts, including the total base count for each file, GC composition, and the numbers and the frequencies of the homopolymer tracts of different lengths. Poly uses a moving window of 1 bp in length to differentiate tracts and spacers, taking into account the tags used to prevent the artifactual concatenation. These data and additional information are kept as data objects in the program and can be manipulated in various ways.

Poly calculates the observed tract frequency of base *i*, , of length N by the formula:



where  is the number of observed tracts of base *i *at length *N *contained in each sequence and *l*_*seq *_is the total length of the sequence (total base count) in which those tracts were counted.

Using the relationship:



the  of tracts of length N can be related to N and P'. The parameter P' is the inverse of the frequency, *f*_*slope*_, of the tract base *i *in the particular genome compartment and is determined from the slope [-log(P')] of an eqn. [1b] plot. The *f*_*slope *_quantity, which represents an effective base frequency for that DNA, can be determined for a given set of data, and then compared to the real frequency for that base occurring in the sequences being examined, as we have previously described [1].

The expected frequency, , of a homopolymer tract of length N randomly occurring is calculated by the formula:



where base frequency  is the fractional base composition of the tract base *i *within the DNA for that file, and N is the tract length.

The level of tract representation for base *i *is then calculated as the ratio of observed to predicted frequencies, defined as "Representation"(*R*):



Values larger than 1 indicates base *i *tracts are "over-represented", while values less than 1 indicate tracts are "under-represented". The log(*R*) verses N plots presented in Figure 6 were generated using the program Gnuplot [38,65]. The values of N at 0.3 to 1.0 of the log(*R*) were found using linear interpolation of the data. We term these N values, *thresholds*, corresponding to particular enrichments of tract occurrence.

The maximum expected length of a homopolymer tract of base *i*, , given the base composition of the entire sequence, , is calculated by the formula:



The length of a given homopolymer tract can then be compared to its expected length by taking the ratio of the longest observed length, , to the longest expected length, . This parameter is defined as "Proportion (*P*)". Thus, we have the formula:



*P *values larger than 1 are called "over-proportional" and P values less than 1 are "under-proportional". *P *represents the tract length comparison on the x-axis of Figure 8, which is complementary to the parameter *R *for tract frequency comparisons on y-axis. They are both important parameters for the evaluation of the frequency and length distributions of any tracts.

## Authors' contributions

YZ carried out the sequence data collection, clean-up, and analysis, and drafted the major part of the manuscript. JWB contributed both major programs Meltsim and Poly in this study, and also participated in analyzing data and drafted part of the manuscript. KAM conceived the study, participated in its design and coordination and revised and finalized the manuscript in all its revisions. All authors read and approved the final manuscript. The authors acknowledge the contribution from a reviewer of the concepts of retroelement insertion and differential polymerase selectivity as potential origins for the higher A & T tract frequencies than G&C tract frequencies that we describe here.
